# View-Angle Tilting and Slice-Encoding Metal Artifact Correction for Artifact Reduction in MRI: Experimental Sequence Optimization for Orthopaedic Tumor Endoprostheses and Clinical Application

**DOI:** 10.1371/journal.pone.0124922

**Published:** 2015-04-24

**Authors:** Pia M. Jungmann, Carl Ganter, Christoph J. Schaeffeler, Jan S. Bauer, Thomas Baum, Reinhard Meier, Mathias Nittka, Florian Pohlig, Hans Rechl, Ruediger von Eisenhart-Rothe, Ernst J. Rummeny, Klaus Woertler

**Affiliations:** 1 Department of Radiology, Klinikum rechts der Isar, Technische Universitaet Muenchen, Ismaninger Strasse 22, 81675, Munich, Germany; 2 Musculoskeletal Imaging, Kantonsspital Graubuenden, Loestrasse 170, CH-7000, Chur, Switzerland; 3 Department of Neuroradiology, Klinikum rechts der Isar, Technische Universitaet Muenchen, Ismaninger Strasse 22, 81675, Munich, Germany; 4 Siemens AG, Healthcare Sector, Allee am Roethelheimpark 2, 91052, Erlangen, Germany; 5 Department of Orthopaedic Surgery, Klinikum rechts der Isar, Technische Universitaet Muenchen, Ismaninger Strasse 22, 81675, Munich, Germany; Shenzhen institutes of advanced technology, CHINA

## Abstract

**Background:**

MRI plays a major role in follow-up of patients with malignant bone tumors. However, after limb salvage surgery, orthopaedic tumor endoprostheses might cause significant metal-induced susceptibility artifacts.

**Purposes:**

To evaluate the benefit of view-angle tilting (VAT) and slice-encoding metal artifact correction (SEMAC) for MRI of large-sized orthopaedic tumor endoprostheses in an experimental model and to demonstrate clinical benefits for assessment of periprosthetic soft tissue abnormalities.

**Methods:**

In an experimental setting, tumor endoprostheses (n=4) were scanned at 1.5T with three versions of optimized high-bandwidth turbo-spin-echo pulse sequences: (i) standard, (ii) VAT and (iii) combined VAT and SEMAC (VAT&SEMAC). Pulse sequences included coronal short-tau-inversion-recovery (STIR), coronal T1-weighted (w), transverse T1-w and T2-w TSE sequences. For clinical evaluation, VAT&SEMAC was compared to conventional metal artifact-reducing MR sequences (conventional MR) in n=25 patients with metal implants and clinical suspicion of tumor recurrence or infection. Diameters of artifacts were measured quantitatively. Qualitative parameters were assessed on a five-point scale (1=best, 5=worst): “image distortion”, “artificial signal changes at the edges” and “diagnostic confidence”. Imaging findings were correlated with pathology. T-tests and Wilcoxon-signed rank tests were used for statistical analyses.

**Results:**

The true size of the prostheses was overestimated on MRI (P<0.05). A significant reduction of artifacts was achieved by VAT (P<0.001) and VAT&SEMAC (P=0.003) compared to the standard group. Quantitative scores improved in the VAT and VAT&SEMAC group (P<0.05). On clinical MR images, artifact diameters were significantly reduced in the VAT&SEMAC-group as compared with the conventional-group (P<0.001). Distortion and artificial signal changes were reduced and diagnostic confidence improved (P<0.05). In two cases, tumor-recurrence, in ten cases infection and in thirteen cases other pathologies were diagnosed.

**Conclusions:**

Significant reduction of metallic artifacts was achieved by VAT and SEMAC. Clinical results suggest, that these new techniques will be beneficial for detecting periprosthetic pathologies during postoperative follow-up.

## Introduction

Preoperative chemotherapy and the development of specific endoprostheses with stronger materials and individual implants have enabled limb-sparing surgery with a good functional outcome instead of amputation [1–5]. However, there are more potential complications with limb salvage than with amputation or rotation plasty [6]. For detection of tumor recurrence, infection, wound necrosis, nerve palsy or prostheses-related pathologies such as loosening, foreign-body reaction or implant failure [7], MR imaging plays a major role after limb salvage surgery [8–11]. However, implants can cause significant metal-induced distortion of magnetic-field homogeneity, leading to errors in slice excitation and spatial encoding. While signal loss due to T2* shortening can be successfully compensated by using spin echoes (SE), spatial distortions during slice-selective excitation and signal readout remain a fundamental problem. Within certain limitations, defined by the available hardware, the allowed specific absorption rate (SAR) and minimum requirements for the signal-to-noise ratio (SNR), spatial distortion can be addressed by employing high bandwidths for excitation and readout. By using stronger gradients, susceptibility due to field inhomogeneities is reduced. In the vicinity of metallic implants, however, these generic methods most often fail to provide sufficient artifact suppression [12–15]. Fortunately, more specific techniques have become available, which are compatible with the methods discussed above.

In-plane displacement in the readout-encoding direction within an excited slice can be corrected by the ‘view angle tilting’ (VAT) technique [16], which applies an additional gradient in slice-encoding direction during signal readout. To reduce blurring, which is increased by VAT, thin slices and high readout bandwidths are mandatory, causing a trade-off with spatial coverage and SNR, respectively [17].

Through-plane distortions, i.e. non-planar slice excitations, can be corrected with a technique, dubbed ‘slice-encoding metal artifact correction’ (SEMAC) [18], by applying additional phase encoding gradients perpendicular to the slice (z-direction) prior to signal readout. Assuming that through-plane distortions of the excited slices can be limited to a block of width SES x slice thickness (SES = number of slice encoding steps) centered on the nominal slice location, a number of SES scan repetitions is needed in order to perform the SEMAC phase encoding. Unlike VAT, SEMAC increases the acquisition time and requires additional post-processing.

Although VAT and SEMAC appear to be useful for metal artifact reduction on MR images after total hip replacement [19] or total knee replacement [20], applicability for large tumor endoprostheses with massive material has not been evaluated so far. A major challenge is the need of high numbers of slices, large fields of view (FOV) and individual adjustment for each patient. Further, clinical benefits of the new sequences in patients with metal implants and suspicion of tumor-recurrence or periprosthetic infection have not been demonstrated so far.

The purpose of this study was (i) to show, that the new metal reducing sequences are applicable with reduced numbers of slice encoding steps (SES), a large FOV and a high number of slices for MR imaging of orthopaedic tumor endoprostheses, (ii) to evaluate quantitatively and qualitatively the benefit of VAT and SEMAC for MR imaging of large-sized orthopaedic tumor endoprostheses in an experimental model and (iii) to demonstrate the benefit of VAT and SEMAC in comparison to previously applied metal artifact reducing MR sequences in the evaluation of periprosthetic soft tissue abnormalities.

## Material and Methods

### Experimental setting

**Fig 1:**
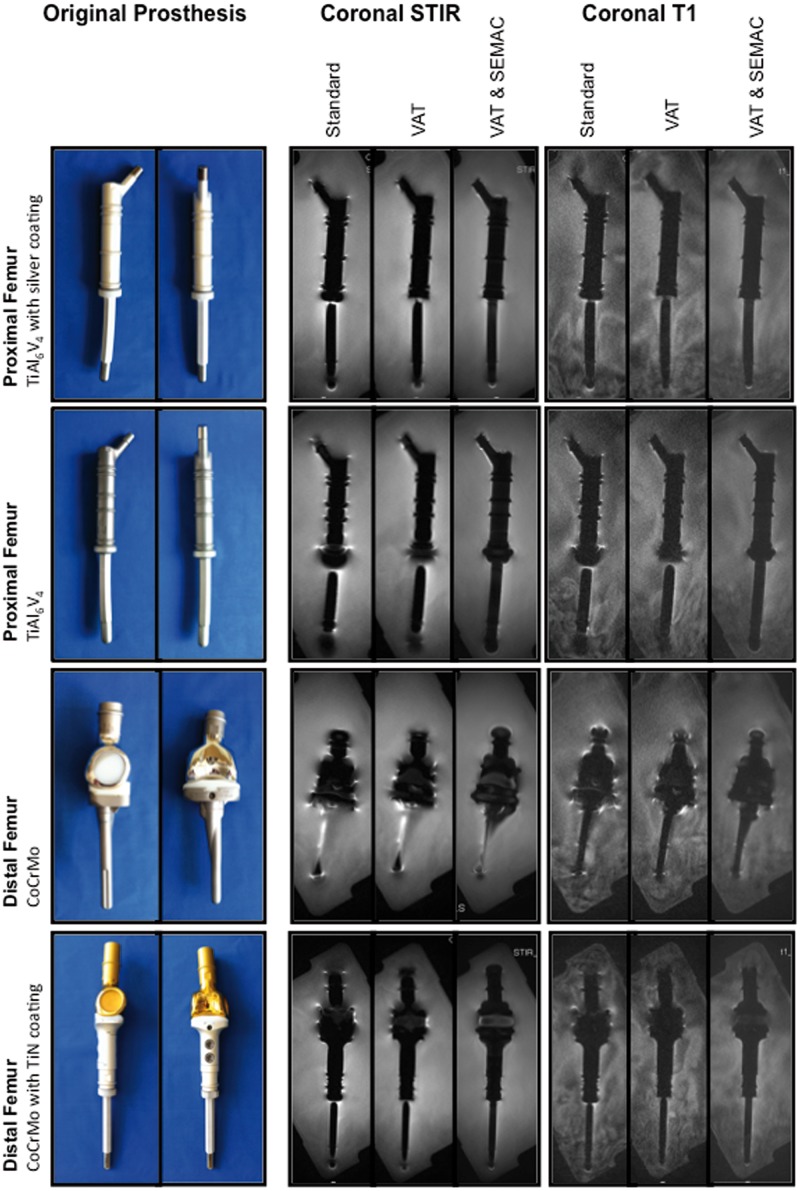
Experimental model. Tumor endoprostheses were embedded in water. Specifications and materials are indicated on the left. Central slices of coronal STIR and coronal T1-w MR pulse sequences are presented for each technique (standard; VAT, view-angle tilting; VAT&SEMAC, view-angle tilting and slice-encoding metal artifact correction).

In an experimental model, four different commercially available orthopaedic tumor endoprostheses for replacement of large bone and joint defects in the lower limbs were assessed (MUTARS, implantcast GmbH, Buxtehude, Germany; Fig 1): (i) Silver-coated titanium (TiAl_6_V_4_) proximal femur replacement with a cemented intraosseous shaft; (ii) Titanium proximal femur replacement with a cementless intraosseous shaft; (iii) Cobalt-chromium (CoCrMo) distal femur replacement attached to a cemented proximal tibia replacement (Cobalt-Chromium) with an interposed polyethylene component; (iv) Titanium nitride (TiN)-coated, CoCrMo distal femur replacement attached to a silver coated titanium proximal tibia implant with a cementless intraosseous shaft and an interposed polyethylene component. Tumor endoprostheses were individually embedded in water, which surrounded the prostheses >10mm in every direction.

### Magnetic resonance (MR) Sequences

MR imaging was performed on a 1.5T system (Magnetom Avanto, Siemens AG, Erlangen, Germany). Experimental scans were performed with a six-channel body phased array coil anteriorly and two spine coil clusters (three channels each) posteriorly. For clinical scans, depending on the localization of the tumor the body coil or dedicated surface coils were used. A work-in-progress software package including turbo spin echo (TSE) pulse sequences, allowing for more flexible adjustment of RF and readout bandwidths and optional application of VAT and SEMAC, was provided by Siemens AG (Siemens AG, Healthcare Sector, Erlangen, Germany).

### Experimental MR scans

Pulse sequences comprising short-tau-inversion-recovery (STIR), T1-weighted (w) and T2-w contrast were adapted for our purposes by increasing the number of slices and enlarging the FOV. Reduction of SES from 10 to 8, 6, 4, 2 and 0 (0 is equivalent to the VAT group) was evaluated for coronal T1-w pulse sequences and findings were confirmed for other prostheses and pulse sequences. MR scans were acquired in three different study groups (Table 1). In the first group, images were acquired by using standard high-bandwidth techniques without application of VAT or SEMAC (otherwise identical sequences; standard group). In the second group VAT was applied (VAT group). In the third group both, VAT and SEMAC with 6 SES were used (VAT&SEMAC group). While SEMAC profits from increasing the number of SES, the direct proportionality between acquisition time and SES is a nontrivial issue. To arrive at clinically reasonable scan durations, the available range of conventional acceleration strategies (increased readout and RF bandwidths, high turbo factors, parallel imaging) has to be exploited. Coronal images were acquired in all three groups, whereas transverse images were acquired in the VAT group and the standard group only. With the exception of transverse coverage of large-sized tumor prostheses, which require a particularly large number of slices, clinically acceptable scan times could be reached in combination with SEMAC.

**Table 1:** MR pulse sequences, experimental.

Pulse sequence	Coronal STIR	Coronal T1	Transverse T2	Transverse T1
**Repetition time (RT; msec)**	7600	546	6810	606
**Echo time (ET; msec)**	40	6.3	576	5.4
**Slice thickness (mm)**	4	4	4	4
**Refocusing flip angle**	150°	140°	136°	120°
**Field of view (FOV; mm)**	500	500	220	220
**Matrix**	256x265	256x256	256x256	256x256
**Bandwidth (Hz/ Pixel)**	651	651	574	610
**Echo train length**	23	7	21	7
**Inversion time (msec)**	145	-	-	-
**Number of slices**	25	27	40	40
**Slice-encoding steps (SES)**				
**SEMAC group**	6	6		
**VAT group, Standard group**	0	0	0	0
**Acquisition time (min)**				
**SEMAC group**	4:43	3:49	4:41	4:55
**VAT group, Standard group**	0:55	0:41		

VAT, view-angle tilting. SEMAC, slice-encoding metal artifact correction. 1cm^2^ = 100mm^2^.

### Clinical example

Exemplarily, VAT&SEMAC was applied in a patient with a large tumor endoprosthesis during routine follow-up MR imaging. MR images were performed in a young, female patient (15 years old) after resection of an osteosarcoma grade III at the left distal femur and implantation of a combined femoral and tibial replacement. Pulse sequences included a coronal STIR sequence (RT, 9000msec; ET, 39msec; slice thickness, 3.5mm; refocusing flip angle, 150°; FOV, 48cm^2^; matrix, 448; bandwidth, 657Hz/Pixel; inversion time, 145msec; number of slices, 30; SES, 6; acquisition time, 6:29min) and a coronal T1-w sequence without and with contrast application (RT, 716msec; ET, 5.7msec; slice thickness, 3.5mm; refocusing flip angle, 140°; FOV, 48cm^2^; Matrix, 512; Bandwidth, 651Hz/Pixel; Echo-spacing, 5.72msec; number of slices, 30; SES, 6; acquisition time, 6:56min).

### Study population

In the following, comparison of VAT&SEMAC and conventional metal artifact reducing MR sequences (conventional) was performed. Twenty-five consecutive patients, referred from the orthopaedic department with metal implants and with clinical suspicion of local tumor recurrence or periprosthetic infection, were prospectively included in the present study. Written informed consent was obtained from all patients. The study was reviewed and approved by the local institutional review boards (Ethikkommission der Fakultaet fuer Medizin der Technischen Universitaet Muenchen, Germany). The study has been conducted according to the principles expressed in the Declaration of Helsinki. Exclusion criteria included general contraindications for MR imaging.

### Clinical MR examinations

VAT&SEMAC-techniques and conventional techniques were compared for coronal STIR (n = 19) and coronal contrast enhanced T1-w sequences (n = 19). All MR sequences were adjusted for each individual patient, based on the experimental MR protocol. MR parameters are presented in Table 2.

**Table 2:** Exemplary clinically applied MR pulse sequences.

Pulse sequence	Coronal STIR Conventional	Coronal STIR VAT&SEMAC	Coronal T1 Conventional	Coronal T1 VAT&SEMAC
**Repetition time (RT; msec)**	5350	9150	500	716
**Echo time (ET; msec)**	60	43	15	5.7
**Slice thickness (mm)**	5	3.5	5.	3
**Refocusing flip angle**	180	150	180	140
**Field of view (FOV; mm)**	400	400	400	400
**Matrix**	384x384	238x384	517x640	410x512
**Bandwidth (Hz/ Pixel)**	766	651	710	651
**Echo train length**	7	23	5	7
**Inversion time (msec)**	155	145	-	-
**Number of slices**	20	28	20	28
**Slice-encoding steps (SES)**	-	6	-	6
**Acquisition time (min)**	4:13	5:40	5:13	6:56

VAT, view-angle tilting. SEMAC, slice-encoding metal artifact correction. 1cm^2^ = 100mm^2^.

### Histopathology

By means of digital medical records histopathologic reports and microbiology reports were retrospectively analyzed in those patients that had undergone biopsy or surgery following the MR examination due to suspicion of tumor recurrence or periprosthetic infection.

### Quantitative Analysis

**Fig 2:**
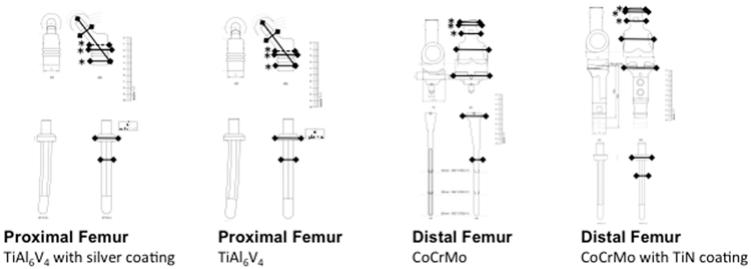
True diameters of orthopaedic tumor endoprostheses. True diameters were measured at six standardized spots as indicated and confirmed on original sketches (continuous lines). Asterisks indicate three standardized spots that were used for comparisons of different implant materials; broken lines indicate diameters that were additionally measured for this purpose.

In the experimental model, for each endoprosthesis, objective quantitative measurements of artifact diameters were performed by two investigators in consensus (P.M.J. and C.J.S.). At six standardized levels, as indicated in Fig 2, artifact diameters (including signal loss and signal pile-up) were compared to true diameters of the prostheses using a vernier capiler. These measurements were confirmed on original sketches of the tumor endoprostheses provided by the manufacturer (implantcast GmbH, Buxtehude, Germany; Fig 2). At three levels (marked with asterisks in Fig 2), real diameters were identical for different implant materials and different coatings, allowing a comparison of artifact sizes for these. On clinical MR images of patients quantitative artifact diameters were measured at four randomly selected spots for each implant.

### Qualitative MR Analysis

Qualitative evaluation of MR images was performed on picture archiving communication system (PACS) workstations (Easy Vision, Philips, Best, The Netherlands). For MR images from the experimental model, evaluation was performed separately by two radiologists (P.M.J. and C.J.S., 5 and 10 years of experience). In case of discordance, consensus readings by both radiologists and an additional independent musculoskeletal radiologist (J.S.B., 12 years of experience) were commenced. Clinical scans were read out in consensus by two radiologists (P.M.J. and R.M. 5 and 8 years of experience). In case of discordance, consensus readings by both radiologists and an additional independent musculoskeletal radiologist (C.J.S., 10 years of experience) were commenced.

Spatial-frequency inhomogeneity-induced susceptibility artifacts were assessed subjectively on a five-point scale (1 = minimal artifacts; 5 = maximum artifacts) [12]:

1. “Geometric image distortion” with anatomic displacement, describing how accurate the true geometric shape of the prosthesis was depicted on the MR image (geometric distortion). This parameter mainly described through-plane image distortion, but was also affected by in-plane distortion.
2. “Artificial signal changes” with signal loss or signal pile-up in the immediate vicinity of the prostheses, especially at the edges, intersections and interfaces between different implant components (signal changes). The parameter”artificial signal changes” implicated both in-plane and through-plane distortion.

For clinical scans, the images were read out diagnostically. Additionally, the parameters “diagnostic confidence” and “spacial blurring" were documented on a five-point scale (1 = best, 5 = worst). Tumor recurrence was diagnosed, if a newly developed mass was identified, that exhibited the same MR characteristics as the primary lesion. Periprosthetic infection was diagnosed, if intense edemateous soft tissue swelling with marked contrast enhancement was depicted; additional loosening of the implant was suspected in case of periprosthetic fluid collections. Abscess formation was diagnosed, if a well defined fluid collection with a thick contrast-enhancing wall and surrounding soft-tissue edema was seen. In contrast, seroma was defined as a localized fluid collection without or with internal structures surrounded by a smooth lining, which exhibited low T2-weighted signal intensity and little to moderate contrast enhancement in the absence of adjacent soft tissue edema. Bursitis was suspected, if a lobulated fluid collection was seen in a typical anatomic location. Bone infarction was defined as a serpingious area within bone marrow, which exhibited a double-line sign and internal fat signal.

### Reproducibility measurements

For qualitative measurements, all experimental MR scans were graded twice for each parameter by two radiologists (P.M.J. and C.J.S.) on two separate occasions with an interval of four weeks in-between the two time points. Inter- and intra-observer agreement was calculated using linear-weighted Cohens Kappa’s values [21]. Reproducibility for quantitative diameter measurements was determined in a sample of 18 randomly selected measurements. Each level was measured three times by two investigators (P.M.J.; C.J.S.). Reproducibility errors were calculated as the root mean square error (ms) and as the root mean square error coefficient of variation (%) [22].

### Statistical analysis

Statistical processing was performed with SPSS version 17.0 (SPSS Institute, Chicago, IL, USA; P.M.J., T.B.). Differences between groups were assessed via T-tests (quantitative assessment) and Wilcoxon-signed rank tests (qualitative assessment). Results are reported as mean ± standard error of the mean (SEM). Mean differences±SEM and lower 95% Confidence Interval (CI) and upper 95%CI were calculated for intergroup comparisons. Differences with a P<0.05 were considered as statistically significant.

## Results

### MR Sequences

**Fig 3:**
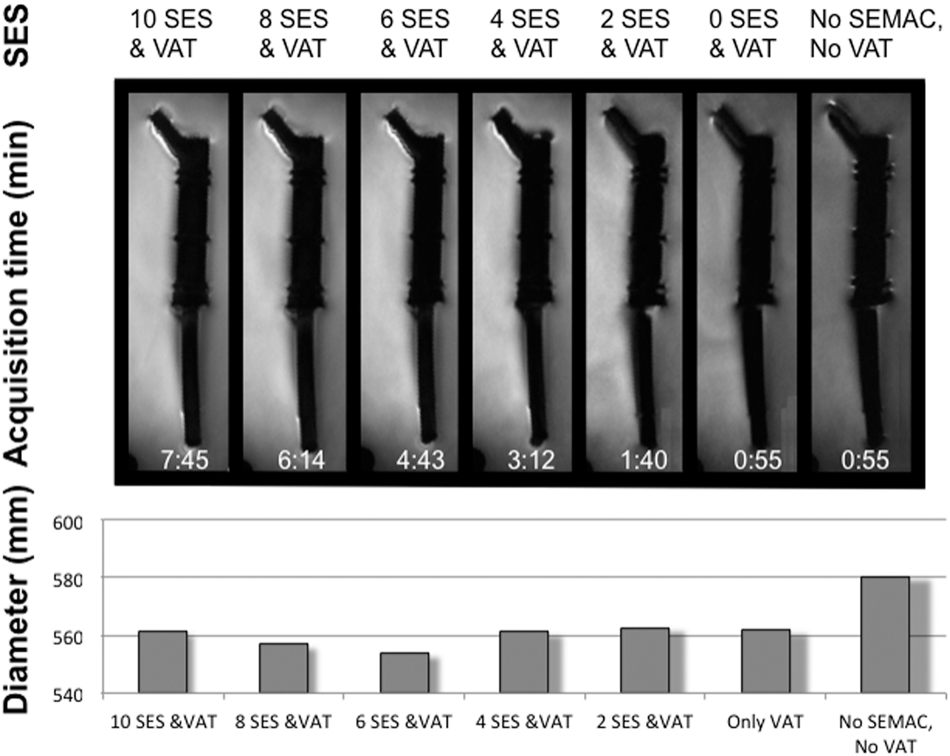
Reduction of slice-encoding steps (SES) for slice-encoding metal artifact correction (SEMAC). SES were reduced from 10 to 0 and assessed quantitatively and qualitatively. No SEMAC is equivalent to 0 SES. Acquisition time for all SES values is shown in the images. For qualitative evaluation, artifact diameters (mm) were measured on all MR images. VAT, view-angle tilting. SEMAC; slice-encoding metal artifact correction.

The number of SES for SEMAC was reduced with respect to the original MR protocol with 12 SES (Fig 3). The artifact diameter was significantly larger in the standard group than in all other groups (mean difference±SEM from 1.8±0.7mm to 2.6±1.0mm; P<0.05). Diameters in the groups with different SES were not statistically significance. Subjective qualitative evaluation showed a continuous reduction of artifacts with increasing SES until 6 SES (Fig 3).

### Experimental quantitative MR analysis

**Fig 4:**
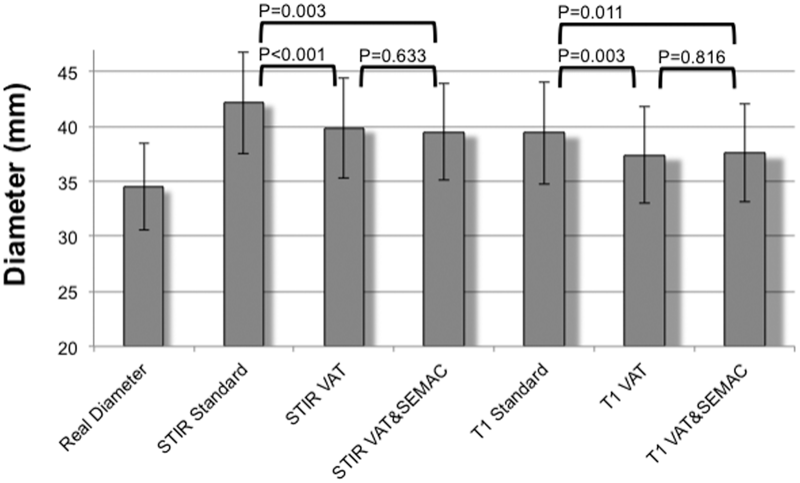
Quantitative results for coronal MR pulse sequences (STIR and T1-w sequences). Diameter measurements were performed at six standardized levels on all MR images and compared to true diameters of the tumor endoprostheses. Study groups were a standard group (Standard) without application of VAT (view-angle tilting) or SEMAC (slice-encoding metal artifact correction) but otherwise identical pulse sequences, a group where VAT was applied (VAT; no SEMAC) and a group where both techniques, VAT and SEMAC, were applied (VAT&SEMAC). Mean values ±SEM are presented. True diameters were significantly smaller than artifact diameters in all groups (P<0.05).

Mean true diameter±SEM at the measured levels of the tumor prostheses was 34.7±4.0mm, which was significantly smaller than artifact diameters on all MR images (P<0.05; Fig 4; Table 3). The largest artifact diameters were measured on standard coronal STIR images (42.2±4.7mm; P<0.001). Artifacts in the VAT and in the VAT&SEMAC group had a diameter of 39.9±4.6mm (P<0.001) and 39.5±4.4mm (P = 0.003). Artifacts were significantly smaller on T1-w images compared to STIR images (P<0.05). On T1-w images the mean artifact diameter was 39.4±4.7mm in the standard group, 37.4±4.5mm in the VAT group (P = 0.003) and 37.6±4.5mm in the VAT&SEMAC group (P = 0.011). On transverse T2-w images, mean artifact diameter was 48.1±4.0mm in the standard group and 45.6±3.0mm in the VAT group (P = 0.016; T1-w images, 45.2±3.6mm versus 42.0±3.2mm, P = 0.031). On coronal STIR images, artifacts produced by implants made from TiAl_6_V_4_ were smaller than artifacts produced by CoCrMo implants (29.4±0.4mm versus 30.6±0.7mm; P = 0.002). For coronal T1-w images this difference was smaller (TiAl_6_V_4_ versus CoCrMo, 28.7±0.3mm versus 29.5±0.7mm; P = 0.047). There was no significant difference between artifacts produced by proximal femur prostheses (TiAl_6_V_4_) with and without silver coating (P>0.05; STIR, P = 0.957) or between artifacts produced by distal femur prostheses (CoCrMo) with and without TiN coating (P>0.05; STIR, P = 0.464).

**Table 3:** Quantitative measurements, experimental.

	Coronal STIR VAT	Coronal STIR VAT& SEMAC	Coronal T1VAT	Coronal T1VAT&SEMAC	Transverse T2 VAT	Transverse T1 VAT
**Mean Difference vs. "Standard”(mm)**	-2.3	-2.7	-2.0	-1.9	-2.5	-3.2
**SEM**	0.5	0.8	0.6	0.7	1.0	1.4
**Lower 95% CI**	-1.3	-1.0	-0.8	-0.5	-0.5	-0.3
**Upper 95% CI**	-3.3	-4.3	-3.2	-3.2	-4.5	-6.1
**P-value**	<0.001	0.003	0.003	0.011	0.016	0.031

Reduction of artifacts in the VAT group and the VAT&SEMAC group as compared to the standard group for each pulse sequence. SEM, standard error of the mean; CI, confidence interval; P, P (t-test).

### Experimental qualitative MR analysis

**Fig 5:**
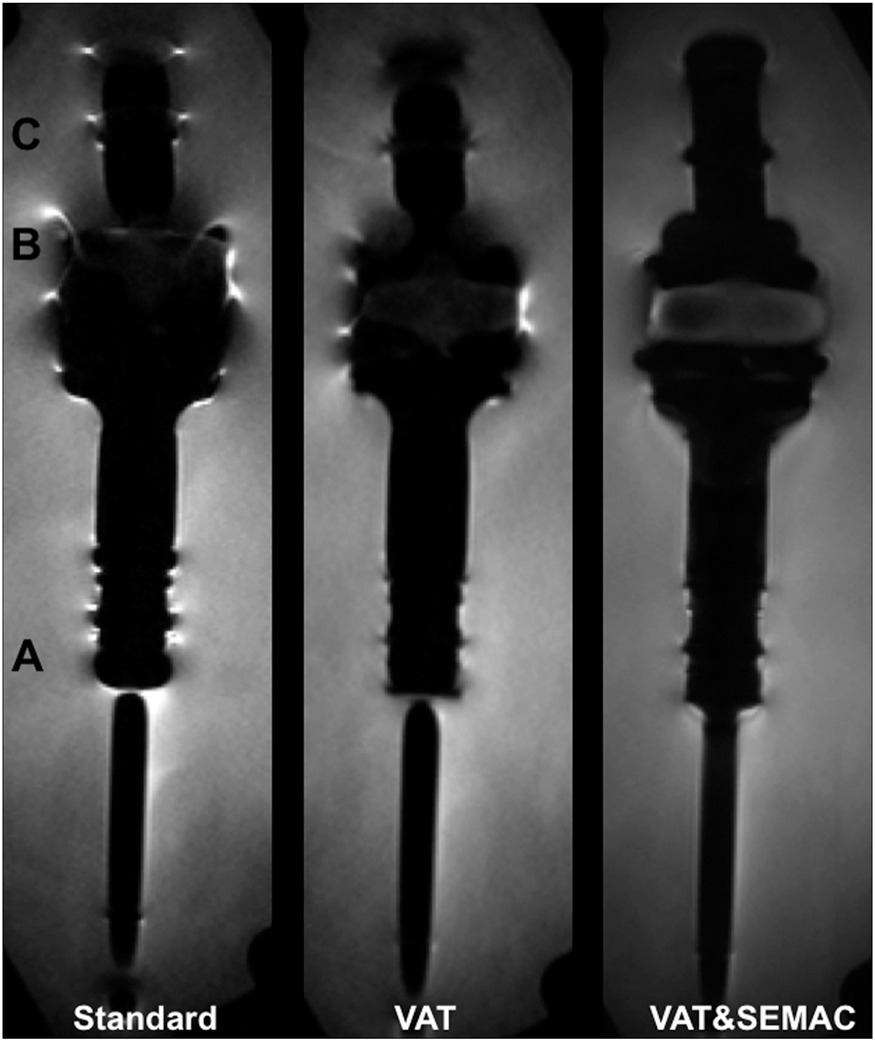
Coronal STIR images of one tumor endoprosthesis are presented for all groups (standard group; VAT (view-angle tilting) group; VAT&SEMAC (view-angle tilting and slice-encoding metal artifact correction) group). Exemplarily, “A” indicates the interface between the collar of the stem and the stem of the prosthesis where the qualitative parameter “geometric distortion” was evaluated. “B” indicates the true position of the polyethylene component, which is interposed between the distal femoral component and the proximal tibial component of the endoprosthesis. “C” indicates the signal loss and signal pile-up artifacts at the edges of the prosthesis.

With respect to the qualitative parameter “geometric distortion” combined application of VAT and SEMAC (VAT&SEMAC group) showed the smallest artifacts (STIR, 1.50±0.25, P = 0.002; Table 4). The VAT group showed a mean value of 3.25±0.25 and the standard group showed a mean value of 4.25±0.25. Less distortion was observed on coronal T1-w images as compared to STIR images. On transverse images, distortion was reduced by application of VAT. Distortion was seen at the interface between the collar of the stem and the stem of the prosthesis (Fig 5A) and between the metal and the polyethylene component (“B“). “Artificial signal changes” were reduced by trend on MR images in the VAT group as compared to the standard group (STIR; 3.50±0.25 versus 4.25±0.50 P = 0.058; Table 4). Significant reduction of signal changes was achieved by additional application of SEMAC (2.75±0.25; Fig 5C). Similar results were found for coronal T1-w images.

**Table 4:** Qualitative evaluation, experimental.

	Geometric distortion		Signal changes	
	Mean ± SEM	P-value	Mean ± SEM	P-value
**Coronal STIR Standard**	4.3 ± 0.3		4.3 ± 0.5	
**Coronal STIR VAT**	3.3 ± 0.3	<0.001	3.5 ± 0.3	0.058
**Coronal STIR VAT&SEMAC**	1.5 ± 0.3	0.002	2.8 ± 0.3	0.014
**Coronal T1 Standard**	3.3 ± 0.3		3.5 ± 0.3	
**Coronal T1 VAT**	2.3 ± 0.3	<0.001	2.8 ± 0.5	<0.001
**Coronal T1 VAT&SEMAC**	1.5 ± 0.6	0.006	1.5 ± 0.6	0.060
**Transverse T2 Standard**	4.5 ± 0.3		4.8 ± 0.3	
**Transverse T2 VAT**	3.0 ± 0.4	0.014	3.8 ± 0.5	0.391
**Transverse T1 Standard**	4.3 ± 0.5		4.0 ± 0.4	
**Transverse T1 VAT**	3.0 ± 0.6	0.015	3.3 ± 0.5	0.182

Reduction of artifacts in the VAT group and the VAT&SEMAC group as compared to the standard group for each pulse sequence. Parameters were “geometric distortion” and “signal changes”. SEM, standard error of the mean; CI, confidence interval. SEM, standard error of the mean; CI, confidence interval; P-value, P (Wilcoxon-signed rank test) compared to “standard” for each pulse sequence.

### Clinical example

**Fig 6:**
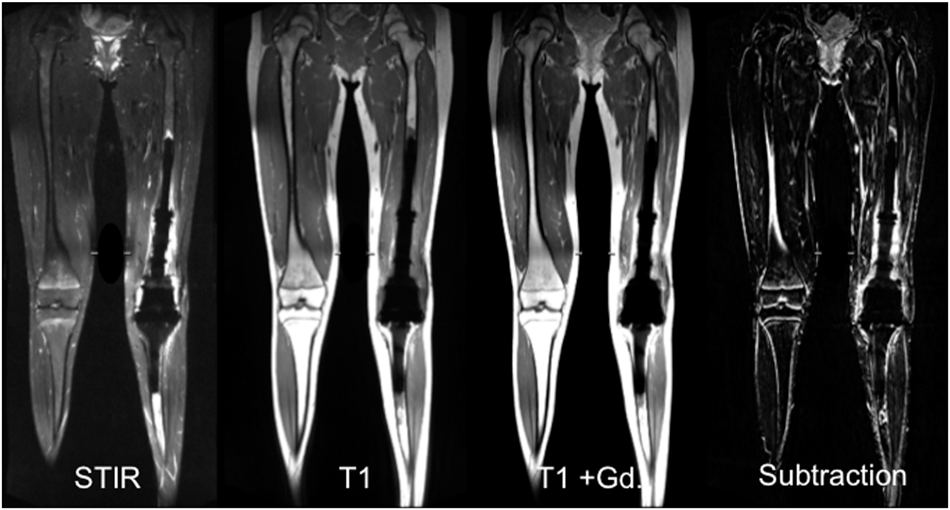
Example of the large size of the field of view. A Large field of view is needed for postoperative follow-up MR imaging in tumor patients. MR imaging was performed in a young, female patient (15 years old) after resection of an osteosarcoma at the left distal femur and implantation of a combined femoral and tibial replacement. Pulse sequences presented include a coronal STIR sequence and a coronal T1-w sequence without and with contrast application and a subtraction image, all using view-angle tilting and slice-encoding metal artifact correction with 6 slice encoding steps.

MR scans of a female patient after resection of an osteosarcoma are presented in Fig 6. Postoperative signal changes of the surrounding tissue, but no suspicious pathologies were detected.

### Clinical results

**Fig 7:**
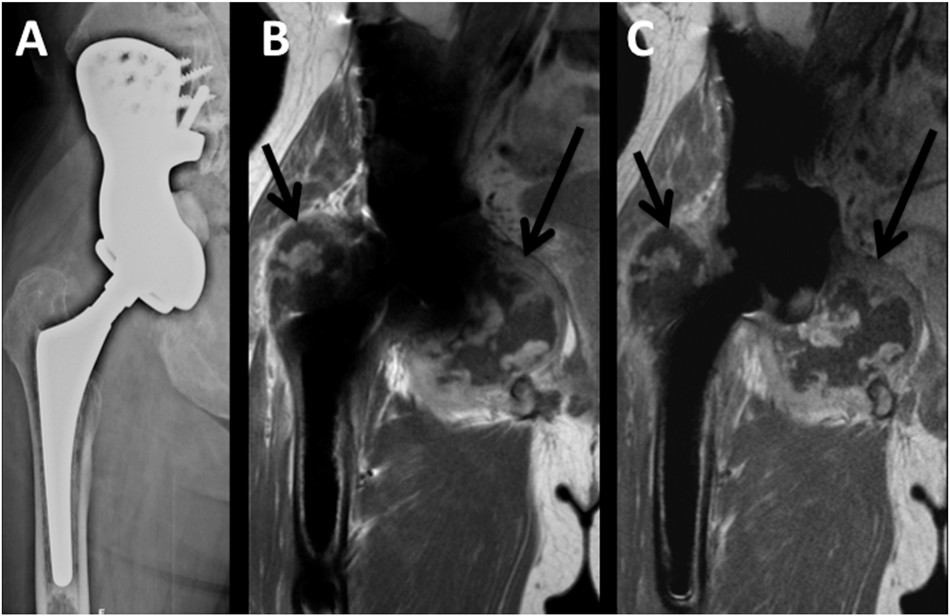
Tumor recurrence. Hip endoprosthesis with tumor recurrence of a chondrosarcoma in a 45 year old male patient. (A) a.p. radiograph; (B) Coronal contrast enhanced T1-w image with conventional artifact reducing MR techniques; (C) Coronal contrast enhanced T1-w image with VAT&SEMAC techniques.

**Fig 8:**
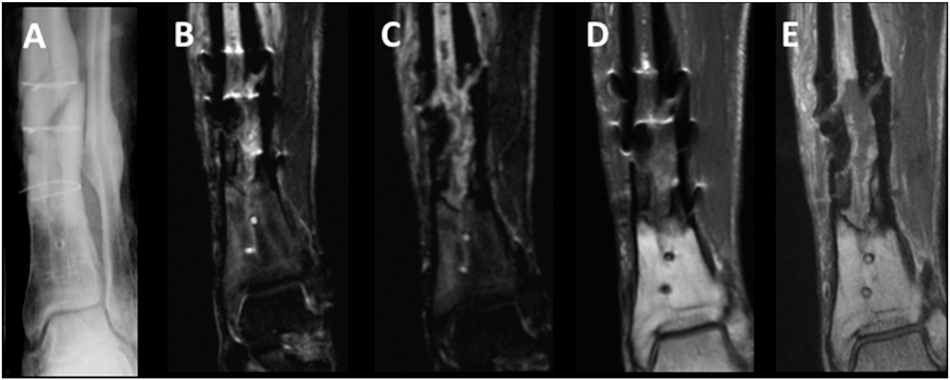
Infection. Posttraumatic pseudarthrosis at the left distal tibia with infection and intramedullary abscess formation in a 27 year old male patient with remaining cerclage fixation. (A) a.p. radiograph; (B) conventional coronal STIR image; (C) VAT&SEMAC coronal STIR image; (D) conventional coronal contrast enhanced T1-w image; (E) VAT&SEMAC contrast enhanced coronal T1-w image.

Twenty-five consecutive patients (13 male, 12 female) with n = 30 metal implants and a mean age ±standard deviation (SD) of 58.5±20.9 years (range, 15–80 years) were prospectively included. If patients had two different metal implants (5/30), the site that presented clinical symptoms was evaluated. Implants were located at the upper extremities (4/30; humerus or radius), the lumbar spine (4/30) or the lower extremity (22/30; proximal femur, knee or tibia). In 12/25 patients infection or tumor recurrence was suspected on MR imaging and in 13/25 patients surgery or biopsy and consecutive pathological and microbiological analysis was performed. On MR imaging, tumor-recurrence was diagnosed in 2/25 cases (n = 1 chondrosarcoma, n = 1 plasmacytoma; pathologically confirmed; Fig 7; Table 5). In one (1/25) case, tumor recurrence of a chondrosarcoma and periprosthetic infection could not be differentiated on MR imaging, and pathology confirmed wear disease with chronic infection without tumor recurrence. Periprosthetic infection was diagnosed in 9/25 cases, (3/9 with abscess formation; 3/9 with loosening of the implant; Fig 8); in all cases pathological analyses confirmed acute or chronic infection. One patient had surgery and presented neither on MR imaging nor in histopathological analyses signs of infection or tumor recurrence. In 12/25 patients other pathologies were diagnosed such as seromas, bursitis or bone infarct.

**Table 5:** Initial diagnoses of the analyzed patient cohort.

Initial diagnosis / Query	Number of patients (n)
Total	25
Suspicion of periprosthetic infect based on clinic and radiographs	7
History of chondrosarcoma	5 ^a^
History of abscess formation	4
History of osteosarcoma	2
Osteolysis on radiographs	2
History of leiomyosarcoma	1
History of ewing sarcoma	1
History of plasmacystoma	1 ^a^
History of renal cell carcinoma	1
History of malignant melanoma & Breast cancer & Chondrosarcoma	1

^a^ Recurrence was diagnosed for one patient with a history of chondrosarcoma and for one patient with a history of plasmacytoma.

### Clinical MR analysis

Artifacts were significantly reduced in the VAT&SEMAC-group as compared with the conventional-group (Figs 7 and 8). For coronal STIR images, the mean difference ±SEM of artifact diameters was 10.2±1.6mm (P<0.001; T1-w, 4.1±0.6mm, P<0.001; Table 6). On images with VAT&SEMAC-techniques, distortion and artificial signal changes were reduced (P<0.001). Although all diagnoses could be suspected on conventional MR images and MR images acquired with VAT&SEMAC techniques by an experienced musculoskeletal radiologist, diagnostic confidence improved significantly for VAT&SEMAC techniques compared to conventional techniques (STIR, 2.1±0.2 vs 4.0±0.3, P<0.001; T1-w, 1.5±0.2 vs 2.8±0.3, P = 0.001; Table 7). “Spacial blurring” however increased with application of VAT&SEMAC.

**Table 6:** Quantitative measurements, clinical.

	Coronal STIR	Coronal T1
**Mean Difference vs. "Conventional”(mm)**	10.7	4.5
**SEM**	1.6	5.9
**Lower 95% CI**	7.1	3.4
**Upper 95% CI**	13.3	5.7
**P-value**	<0.001	<0.001

Reduction of artifacts in the VAT&SEMAC groups as compared to the conventional group for each pulse sequence. SEM, standard error of the mean; CI, confidence interval; P-value, P (t-test) compared to “conventional” for each pulse sequence.

**Table 7:** Qualitative evaluation, clinical.

	Geometric distortion		Signal changes		Diagnostic confidence	
	Mean ± SEM	P-value	Mean ± SEM	P-value	Mean ± SEM	P-value
**Coronal STIR conventional**	4.2 ± 0.2		4.2 ± 0.2		4.0 ± 0.3	
**Coronal STIR VAT&SEMAC**	2.1 ± 0.2	<0.001	2.4 ± 0.2	<0.001	2.1 ± 0.2	<0.001
**Coronal T1 conventional**	3.7 ± 0.2		3.7 ± 0.2		2.8 ± 0.3	
**Coronal T1 VAT&SEMAC**	1.7 ± 0.2	<0.001	2.0 ± 0.2	<0.001	1.5 ± 0.2	0.001

Reduction of artifacts in the VAT&SEMAC group as compared to the conventional group for each pulse sequence. Parameters were “geometric distortion”,”signal changes”, “diagnostic confidence” and “spacial blurring". SEM, standard error of the mean; P-value, P (Wilcoxon-signed rank test) compared to “conventional” for each pulse sequence.

### Reproducibility measurements

For the parameter “geometric image distortion” (“artificial signal changes”), inter-observer kappa was 0.80 (0.61); Intra-observer kappa was 0.90 (0.76) and 0.90 (0.83), respectively. Intra-reader reproducibility for diameter measurements was 0.2mm and 0.2mm (0.68% and 0.64%). Inter-reader reproducibility was 0.3mm (0.96%).

## Discussion

This study analyzed the reduction of susceptibility artifacts, produced by orthopaedic tumor endoprostheses on MR imaging at 1.5 Tesla by application of recently provided metallic-artifact compensation strategies (VAT, SEMAC) in an experimental model. Significant reduction of artifacts was achieved on images of coronal STIR, coronal T1-weighted (w), transverse T1-w and transverse T2-w pulse sequences. While in-plane artifacts were reduced by application of VAT, through-plane artifacts were mainly reduced by additional application of SEMAC. However, on images of all pulse sequences, artifact diameters were still significantly larger than the corresponding true diameters of the prostheses. Additionally, the clinical profit of these sequences for patients with metallic implants and suspicion of tumor recurrence or infection was demonstrated. In comparison to conventional artifact reducing MR sequences, images acquired with VAT and SEMAC techniques showed less artifacts and had better diagnostic quality.

MR imaging represents the standard modality for follow-up of patients with malignant bone tumors at the extremities after surgical excision [8, 23]. Apart from detection of local recurrence [24, 25], several implant-related complications need to be considered during postoperative follow-up, including seroma, hematoma, prosthetic loosening, foreign-body reaction (wear disease) and acute or chronic infection. Regarding the application of metal-artifact-reducing pulse sequences in patients with large tumor prostheses, the particular technical challenge is to obtain a large FOV and a high number of slices, which are mandatory to cover the entire prosthesis, adjacent bone segments, and surrounding soft tissues (Fig 5) [23, 26]. So far, VAT and SEMAC have been applied in situations, where the number of slices and the FOV was comparatively small, e.g. for routine applications such as imaging of hip arthroplasty [19]. A large FOV and high number of slices cause SAR issues and increase the scan time dramatically with its disadvantages of motion artifacts and patient discomfort. Therefore, in our study, metal reducing pulse sequences were tested, that showed a good trade-off between artifact reduction and clinical applicability.

The magnetic-field homogeneity requirements of spectral fat suppression techniques are not compatible with typical inhomogeneities, caused by large metallic implants. For robust fat suppression, STIR pulse sequences had to be used [13, 27, 28], enforcedly accepting well-known drawbacks, such as loss of SNR and inevitable modification of available contrasts. For all groups, images of STIR sequences showed larger artifacts than images of T1-w pulse sequences in our study, confirming previously published findings of other investigators [29].

In the present study, there was no difference in artifact size on MR images of implants with silver or TiN coating as compared to implants without these coating; possibly, because these coatings are only very thin layers. Silver coating is used in patients with a higher risk of prosthetic infection, TiN coating to prevent allergic reactions. A significant difference between artifacts produced by implants made of titanium versus implants made of cobalt-chromium was found. This is in line with previous findings for dental implants and may be explained by ferromagnetic properties of cobalt [29].

Due to significant artifact reduction in the experimental model and proven clinical applicability, VAT&SEMAC techniques were clinically applied and compared to conventional artifact reducing MR sequences. Clinical benefits were demonstrated. Artifacts were reduced significantly and diagnostic performance improved. MR-morphological differentiation between tumor recurrence, abscess formation and wear disease may be simplified. Of note, conventional MR sequences were already optimized for metal artifact reduction. In the study cohort, diagnoses could also be depicted by an experienced radiologist on the previously optimized sequences. However, diagnostic confidence improved significantly if images were acquired with VAT&SEMAC techniques.

For SEMAC, the increase of scan time, caused by the additional slice-phase-encoding procedure, needs to be compensated with conventional acceleration strategies (parallel imaging, partial sampling, increased echo train length), to ensure clinically acceptable scan times. Due to the large variability of implant sizes and materials, in combination with other factors, such as image orientation and patient age, sequence parameters depend to a large extent on the individual situation. Together with the interdependence of specific parameters (RF and readout bandwidth, slice thickness, SES) and their intrinsic trade-offs, sequence optimization becomes a difficult task [28].

Several limitations of this study have to be mentioned. First, artifacts were reduced by the application of different artifact reduction strategies, but despite blinded qualitative and quantitative evaluation, biases were not avoidable. Second, in qualitative analyses, distinct differentiation between through- and in-plane artifacts was not possible. Signal loss, signal pile-up and geometric distortion are influenced by both, through-plane and in-plane artifacts. One major disadvantage of the VAT&SEMAC techniques is an increased blurring of the MR image. Last, there was only a small number of clinical cases, which were scanned using SEMAC and VAT and that were compared with the conventional MR artifact reducing sequences.

In conclusion, optimized pulse sequences, equipped with VAT and SEMAC, were applicable with reduced number of SES, a large FOV and a high number of slices for MR imaging of large-scale tumor endoprostheses and clinical benefits were demonstrated. These sequences reduced metal artifacts significantly in the quantitative and qualitative analyses and thus may facilitate the follow-up of patients who have undergone limb-sparing surgery. Orthopaedic oncology may represent one of the most important clinical applications for these new techniques.
